# Depletion of Myostatin b Promotes Somatic Growth and Lipid Metabolism in Zebrafish

**DOI:** 10.3389/fendo.2016.00088

**Published:** 2016-07-04

**Authors:** Yanping Gao, Ziru Dai, Chuang Shi, Gang Zhai, Xia Jin, Jiangyan He, Qiyong Lou, Zhan Yin

**Affiliations:** ^1^Key Laboratory of Aquatic Biodiversity and Conservation, Institute of Hydrobiology, Chinese Academy of Sciences, Wuhan, China; ^2^University of Chinese Academy of Sciences, Beijing, China; ^3^Key Laboratory of Development and High-Value Utilization of Beibu Gulf Seafood Resource, Guangxi Key Laboratory of Beibu Gulf Marine Biodiversity Conservation, College of Food Engineering, Qinzhou University, Qinzhou, China

**Keywords:** zebrafish, myostatin, myogenesis, lipogenesis, energy metabolism

## Abstract

Myostatin (MSTN) is a negative regulator of myogenesis in vertebrates. Depletion of *mstn* resulted in elevated muscle growth in several animal species. However, the report on the complete ablation of *mstn* in teleost fish has not yet become available. In this study, two independent *mstnb*-deficient mutant lines in zebrafish were generated with the TALENs technique. In the *mstnb*-deficient zebrafish, enhanced muscle growth with muscle fiber hyperplasia was achieved. Beginning at the adult stage (80 days postfertilization), the *mstnb*-deficient zebrafish exhibited increased circumferences and body weights compared with the wild-type sibling control fish. Although the overall total lipid/body weight ratios remained similar between the *mstnb*-deficient zebrafish and the control fish, the distribution of lipids was altered. The size of the visceral adipose tissues became smaller while more lipids accumulated in skeletal muscle in the *mstnb*-deficient zebrafish than in the wild-type control fish. Based on the transcriptional expression profiles, our results revealed that lipid metabolism, including lipolysis and lipogenesis processes, was highly activated in the *mstnb*-deficient zebrafish, which indicated the transition of energy metabolism from protein-dependent to lipid-dependent in *mstnb*-deficient zebrafish. Our *mstnb*-deficient model could be valuable in understanding not only the growth trait regulation in teleosts but also the mechanisms of teleost energy metabolism.

## Introduction

Somatic growth is one of the most important economic traits in fisheries. This polygenic trait is under the influence of multiple physiological pathways regulating energy metabolism and muscle growth (1). Transgenic overexpression of somatotropic genes, such as GH and IGF1, can enhance growth or muscle cell hyperplasia (2, 3). On the other hand, deflated signaling of the suppressors of the somatotropic axis, such as the suppressor of cell signaling 1a (SOCS1a), can enhance somatic growth (4). Application of genetic editing technologies, such as TALENs, CRISPR/Cas9, and transgenic technology, provides a chance to explore insights into somatic growth modulation.

Myostatin (MSTN), also called growth and differentiation factor 8, is generally expressed in skeletal muscle. It is a member of the transforming growth factor β (TGFβ) superfamily. MSTN has been shown to be a negative regulator of satellite cell growth and postnatal myogenesis through the Pax7 signal. Natural mutation of the *mstn* locus resulted in the double-muscle cattle called Belgian Blue and Piedmontese, which are characterized by an increase in muscle mass relative to conventional cattle (5). MSTN-deficient mice exhibited dramatically increased muscle mass, decreased fat deposition, improved insulin sensitivity, enhanced fat oxidation, and increased resistance to obesity (6–8). Similar to MSTNs of other vertebrate species, MSTNs of teleost fish derived from a precursor protein consist of a signal peptide, an N-terminal prodomain, and a C-terminal active domain (9). Zebrafish *mstna* is expressed throughout embryogenesis; however, *mstnb* is active during myogenesis. Therefore, *mstnb* is considered to have important role in postembryonic muscle development (10). Previously, in zebrafish, deflated MSTNb signaling was achieved through morpholino or antisense RNAi (11, 12). Overexpression of dominant-negative form of MSTN through transgenic technology promoted muscle cell proliferation (9, 13). Due to the technical limitation, the phenotype of *mstnb* knockdown zebrafish models was inconspicuous compared with *mstn-*targeting mammalian models. Transgenic expression of *follistatin1* in zebrafish muscle, which could inhibit MSTN signaling, resulted in enhanced muscle hyperplasia (14).

Adipose tissue plays a critical role in regulating energy metabolism and is closely associated with metabolic diseases, such as obesity and diabetes. In mammals, adipose tissue is generally classified into two classes: white adipose tissue (WAT) and brown adipose tissue (BAT). The BAT contains abundant mitochondria and uses lipids for thermogenesis with the unique expression of uncoupling protein 1 (UCP1), which burns chemical energy as heat (15). In WAT, lipid droplets fuse into a larger lipid droplet that is stored as an energy reserve (16). BAT is not present in teleost fish; however, WAT has been reported in common carp (17), Atlantic salmon (18), and zebrafish. WAT is present from 12 days postfertilization (dpf) in the pancreas and in visceral, subcutaneous, and cranial tissues in adult zebrafish (19).

To address MSTNb functions in fish, we generated two *mstnb*-deficient mutant zebrafish strains using TALENs technology. The *mstnb*-deficient zebrafish were indistinguishable from the wild-type zebrafish prior to the juvenile stage. However, after 80 dpf, the mutant fish showed enhanced somatic growth and myofibre hyperplasia. The sizes of the subcutaneous and visceral adipose tissues (VAT) in the *mstnb*-deficient fish decreased, while increased lipid accumulation in muscle tissue was observed. The transcriptional levels of an array of genes in muscle tissue analyzed through quantitative PCR showed that the loss of *mstnb* in zebrafish resulted in enhanced lipid metabolism, including lipolysis and lipogenesis, decreased protein and amino acid degradation, and increased gluconeogenesis. Taken together, enhanced lipid dependence and weakened protein dependence for energy expenditure were adapted in *mstnb*-deficient fish to promote somatic growth during adulthood.

## Materials and Methods

### Zebrafish Husbandry

AB-line zebrafish were maintained on a 14-h light/10-h dark rhythm in circulated water at 28.5°C. The zebrafish were fed newly hatched brine shrimp and TetraMin Tropical Fish food flakes (Tetra, Germany) three times a day. The fat composition in the food supplies is 15 and 8%, respectively (20). Embryos were obtained through natural spawning and cultured at 28.5°C in egg water containing 0.006% ocean salt. The developmental stages of embryos were determined according to hours postfertilization (hpf) at 28.5°C or following the morphological features, as described previously (21). All of the procedures for experimental animal manipulation were approved by the Animal Research and Ethics Committee of the Institute of Hydrobiology of the Chinese Academy of Sciences.

### *Mstnb* Depletion *via* TALENs

**Figure 1:**
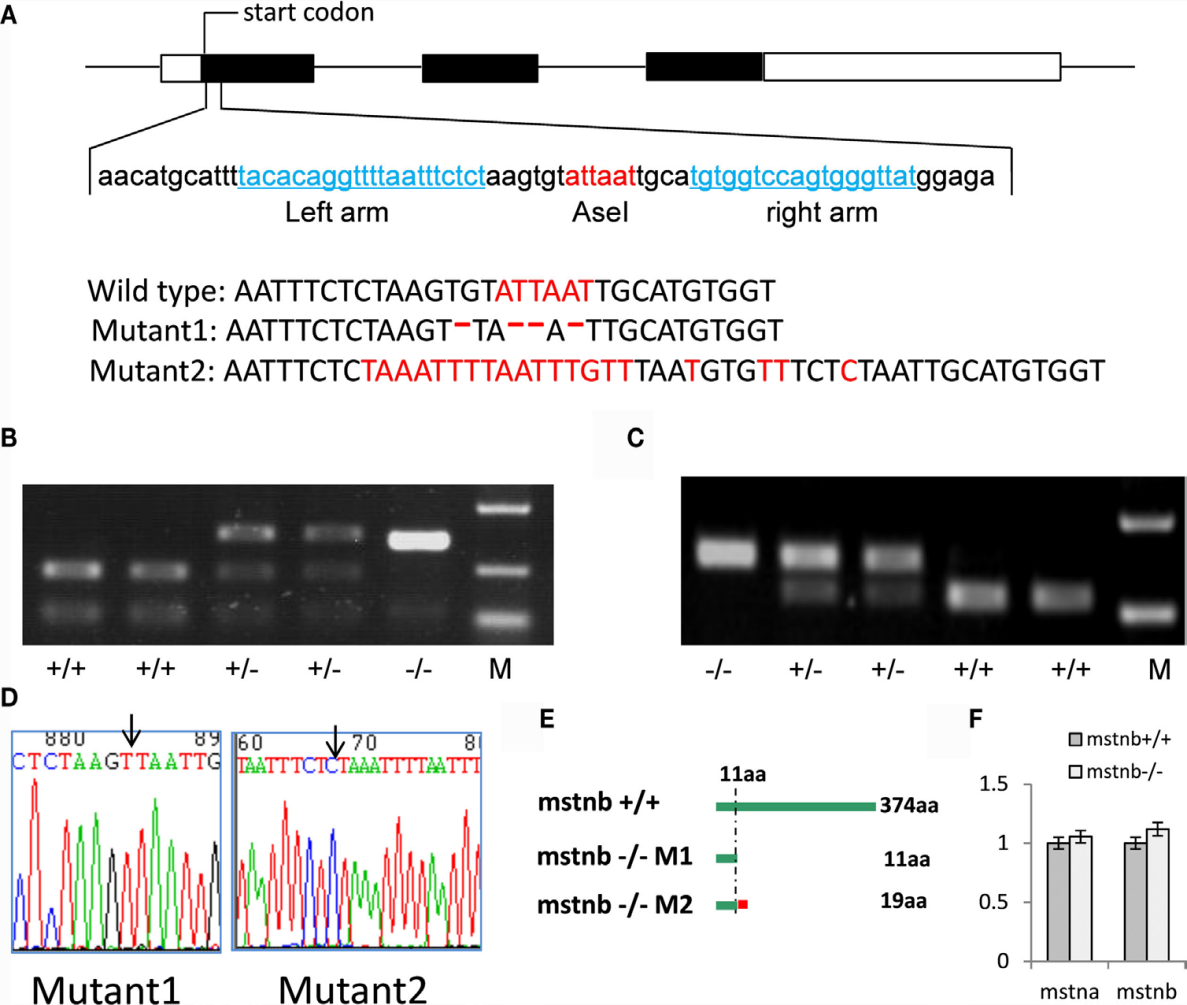
**Disruption of the zebrafish *mstnb* gene by TALENs**. **(A)** The diagram shows the endogenous *mstnb* gene structure, exon: box; the coding region: filled box; and intron: line. The DNA sequences of the left and right arms of the TALENs targeting pair are shown in blue and the *Ase*I digest site within the spacer in red. Lower panel: the alignment of DNA sequences of mutant 1 (M1) and mutant 2 (M2) with wild-type, M1 with a 4 bp deletion, and M2 with a 20-bp insertion. **(B,C)** A representative genotyping for *Ase*I digestion patterning of the PCR products amplified from the genomic DNA from M1 **(B)** and M2 **(C)**; WT represents wild type, +/− represents heterozygous mutant, and −/− represents homozygous mutants. M, DNA molecular marker. **(D)** Sequencing image shows indels of the M1 mutant *mstnb* site (left panel) and of the M2 mutant *mstnb* site (right panel). The arrow head indicates the upstream border of the indels. **(E)** The diagram shows the predicated MSTNb protein from wild-type, M1, and M2 zebrafish, respectively. M1 consists of only 11 aa identical to WT (green); M2 consists of 11 aa identical to WT and 8 miscoding amino acids (red). **(F)** Transcriptional expression levels of myostatinA (mstna) and myostatinB (mstnb). No significant difference was detected between mstnb deficient and wild type control zebrafish.

The construction of the sequence-specific TALENs effector repeats was performed following the procedures described by Huang et al. (22). The four basic single unit vectors, namely NI, NG, NN, and NH, which recognized ATGC, were assembled using *Nhe*I and *Spe*I. To generate capped mRNA-containing DNA-binding TALENs repeats and the *Fok*I endonuclease domain, the TALENs expression vectors were linearized with *Not*I and transcribed using Sp6 mRNA kit (mMessage mMachine kit, Ambion, USA, AM1340). Capped mRNA was injected into wild-type embryos at one- or two-cell stage. Approximately 10 pooled F0 embryos were lysed for genomic DNA isolation, and the target region was then amplified by PCR and digested with *Ase*I (Figure 1). The primers are listed in Table 1. The remaining larvae were raised to adulthood and outcrossed with wild-type fish. The F1 larvae were analyzed by *Ase*I digestion of the targeting region of genomic DNA, and heterozygous *mstnb* transcripts were isolated and sequenced. The F1 heterozygous larvae were raised to adulthood and incrossed to obtain F2 homozygous offspring. The genomic DNA isolated from the fin clip was used for genotyping. Two independent *mstnb-*deficient lines were obtained. Most of the assays were performed with the fish of the mutant 1, unless specifically stated.

**Table 1:** **The primers used in the study**.

Symbol	NCBI accession number	Product length	Melting temp °C	Forward	Reverse
*zebrafish mstnb genotyping*		419	NA	CTGCGAAAGAAGTCCAGCTCT	TTGGAGCCTGCTTGAGTCG
*g6pca1*	NM_001003512.1	104	81.5	ATCGCTGCACCTTACGAGAT	ACCCAGTGAAACACGCTCTC
*scd*	NM_198815.2	143	82.5	CTCACACTCCTCTGGGCTTTT	AAGGCCATGGAGTTTCCGAT
*pck*	NM_214751.1	106	87	GGTCAACAACTGGCCCTGTA	CAGCAGTGAGTTTCCTCCGT
*gk*	NM_001045385.2	102	85	CACCGCTGACCTGCTATGAT	AGTCGGCCACTTCACATACG
*Bckdk*	NM_213060	131	85	CAAAGAGCTGCCTGTCCGTA	AGTCGCTCAGCATGTGGTAG
*Bckdha*	NM_001024419	142	85.5	GCCTGATGTCGATCCGTGTA	CTGGTGCTGTGATGACCGAT
*pax3a*	NM_131277	109	88.5	GCTGGCGGACTCTCCTTATC	CCCAGACTGATGCACTGAGG
*pax7a*	NM_131325	107	86.5	TGACGGGATTCTCGGGGATA	ATGTGGTACGACTGCGTCTC
*Cideb*	NM_001256257.1	124	83.5	GAGCTCAAAGAGAGGGCAGG	AAACAGTGTTGTCCGGCAGA
*fas*	XM_001923608	117	83	CCCTGAAGCATCAGCGTGAA	CTCAGGAAGGCGACCTGAAA
*ACC1*	XM_005165553.2	144	79.5	AACAGGAAACTGTCTGCCCC	CATGCCGTAGTGGTTCAGGT
*Hkdc1*	NM_001115125.1	136	86.5	TCAGCTAATCTGGTGGCTGC	GCCTTTTGGGGTACTGTGGA
*lipea*	NM_001316725.1	91	85	GCGATCCCTCGCAGTTCA	GCTTATGATCCATATCGGACAAC
*LPL*	NM_131127	119	81	ACAATTGACCCAACTGCTGA	GGTTCTTCGAGGGTCCGAAA
*ucp2*	NM_131176	109	87.5	TTCAGAGCTGGTGACGTTCC	TGGTGGAGGCTTTGTTCTCC
*MRF4*	NM_001003982.1	105	84	CCCAGATGGCAGGTCATAGAG	TGGGCTCTTCAGTGGAAATGC
*mstna*	NM_001004122.2	108	82.5	GAACAAGCAAGCAGCGAGAC	AATCTTTGGGCTCAGTGCGA
*MYF5*	NM_131576.1	192	85.5	GCAATACTACAGCCTGCCGAT	CACTGCAAACTGGACACTCCT
*myoD*	NM_131262.2	150	82.5	TTTATGGGCCCAACGTGTCA	TGTGGAAATTCGCTCCACGA
*MyoG*	NM_131006.1	130	81.5	ATTATAGAGCCACCGCCGC	GAGCTATAGGCGGGGACACA

### Histological Analysis, Oil Red O Staining, and Nile Red Staining

The muscle tissue of four animals of each genotype was cryostat sectioned, and the sections were subjected to hematoxylin and eosin staining. The frozen sections were also stained with Oil red O to visualize the fat deposits in the muscle, as described previously (23). The zebrafish were immersed in Ringer’s solution with Nile red for 12 h in the dark, and then the photos were captured at a wavelength of 488 nm for exciting light by an Olympus stereomicroscope.

### Body Fat Ratio Assay

Three adult zebrafish were selected randomly from the *mstnb-*deficient and wild-type control fish. All of the samples were frozen in liquid nitrogen for 72 h. Subsequently, the samples were lyophilized in vacuum drying equipment at −20°C for 24 h. The samples were cut into small pieces, and the weights were recorded before being extracted with methanol and chloroform (volume 3:1) using the Soxhlet extractor. The oil extracted from each fish was weighed. The total oil weight/dry body weight ratios were calculated. The experiments have 4 repeats, and there were 12 individuals taken in analysis. The results were consistent with each other.

### Blood Sugar Measurement in Zebrafish

The OneTouch UltraVue (LifeScan) glucose meter was used for the measurement of blood glucose. The zebrafish were fed regularly with a chow diet and fasted for 12 h prior to blood draws using a heparin-rinsed micropipette tip, as described previously (24). In each time point, there were four *mstnb*-deficient and control zebrafish taken in blood collection, respectively. We used the *T*-test to evaluate the variation of the same time point of the four individual zebrafish. Because the whole blood of one zebrafish was just available for one measure reaction, different group of zebrafish were used to determine the dynamic blood sugar concentration.

### Total RNA Extraction and Gene Expression Levels Quantified with Real-time PCR Analysis

Total RNA was extracted from zebrafish fast muscle tissue using the RNeasy Mini Kit (Qiagen, Germany) and reverse-transcribed with MMLV reverse transcriptase (Thermo, USA). Complementary DNA was diluted 1:50 prior to use. SYBR Green mix was purchased from Transgen Biotech Co., Ltd. (Beijing, China). The primers used for real-time PCR are listed in Table 1. Each pair of primers was verified using regular PCR and electrophoresis until just one single band could be amplified. The PCR products were sequenced for specificity. To analyze the gene expression at larval stages or adult stages, total RNA samples were extracted from at least 40 larvae of each genotype or at least 4 adults of each genotype. The expression level of the *EF-1*α gene was used as the internal control for normalization. The experiments were performed for a minimum of three biological repeats in which new *mstnb*-deficient and control zebrafish were analyzed. The program for real-time PCR was as follows: step 1: 95°C for 15 s; step 2: 95°C for 10 s; step 3: 56°C for 10 s, plate read; step 4: 72°C for 10 s; step 5: repeat step 2 × 39; step 6: 95°C for 10 s; step 7: melting curve, 58–95°C in increments of 0.5°C for 5 s, plate read. The real-time PCR instrument was purchased from Bio-Rad (CFX96 Touch), and the software for calculations was Bio-Rad CFX Manager 3.1.

### Skeletal Muscle Fiber Analysis

For the muscle histological analysis, six individual fish from each group were fixed in formalin for 1 h followed by routine paraffin sectioning and hematoxylin and eosin (H&E) staining. The cross-section at the base of the cloaca was selected to quantify the number of muscle fibers. The muscle fiber area was obtained from digitally imaged serial cross-sections of paraffin-embedded muscle. Individual muscle fibers were outlined, and the cross-sectional area was determined with Image J.

### Western Blot

The protein samples were collected from the whole body skeletal muscle tissue of adult zebrafish. The primary antibodies of anti-S58 (Santa Cruz SC-32733) and anti-GAPDH (Abcam, ab70699) as loading controls were diluted 1:1000 in Can Get Signal primary antibody dilution solution (Toyobo, Japan). There were four biological repeats in this experiment.

### Statistical Analysis

*T*-tests were performed for each experiment. Each result represents the mean of at least three independent experiments. The error bars represent the SDs. The *P* values were calculated and are indicated in the figure legends.

## Results

### Generation of *mstnb* Depletion Lines in Zebrafish

The TALENs-based genome editing technique was used for *mstnb* depletion in zebrafish. Based on the sequence information (Figure 1A), the target site of TALENs was located at the first exon of the gene locus. Both arms of the designed binding arms were 18 bp. The spacer between the two arms was 16 bp in length. The *Ase*I restriction digestion site within the space region could be efficiently used for genotyping. To test the efficiency of the depletion, the targeting region of the *mstnb* locus was amplified from genomic DNA of F0 embryos injected with TALENs mRNAs and *Fok*I nucleases at 3 dpf. Then, the PCR fragment was digested with *Ase*I. The incompletion of digestion by *Ase*I indicated the existence of an indel within the targeting region after application of the depletion procedure, and the rest of the embryos were raised into adulthood. F0 adult fish were then crossed with wild-type fish to obtain F1 embryos, and a pool of F1 embryos from each cross pair was subjected to genotyping by *Ase*I digestion of the PCR products of the targeting region. The F0 founders fish harboring indels in *mtsnb* were used to produce F1 fish. The F1 fish were subjected to genotyping using the genomic DNA extracted from the clip of tail fins at their 30–50 dpf stage. The typical *Ase*I digestion pattern is shown in Figures 1B,C for mutant 1 and mutant 2. The heterozygous F1 fish showed a gel pattern in which half of the fragments were digested, complete digestion for WT, and undigested products of homozygous embryos derived from the heterozygous cross. After sequencing of the *mstnb* locus of homozygous embryos, two independent mutant lines, M1 and M2, were confirmed (Figure 1D) with a 4 bp deletion and 20 bp insertion, respectively (Figure 1A). The putative transcripts from M1 and M2 terminated prematurely to produce a truncated peptide with only 11 and 19 of the N-terminal amino acids identical to zebrafish MSTNb, respectively (Figure 1E). Additionally, we checked the transcriptional expression of *mstna* and *mstnb* to determine the redundant effect to compensate for MSTNb depletion. The results showed that the expression of *mstna* and *mstnb* did not increase significantly (Figure 1F). Therefore, transcriptional compensation of *mstnb* depletion was not observed.

### Muscle Fiber Hypoplasia and Enhanced Body Growth in *mstnb*-Deficient Zebrafish

**Figure 2:**
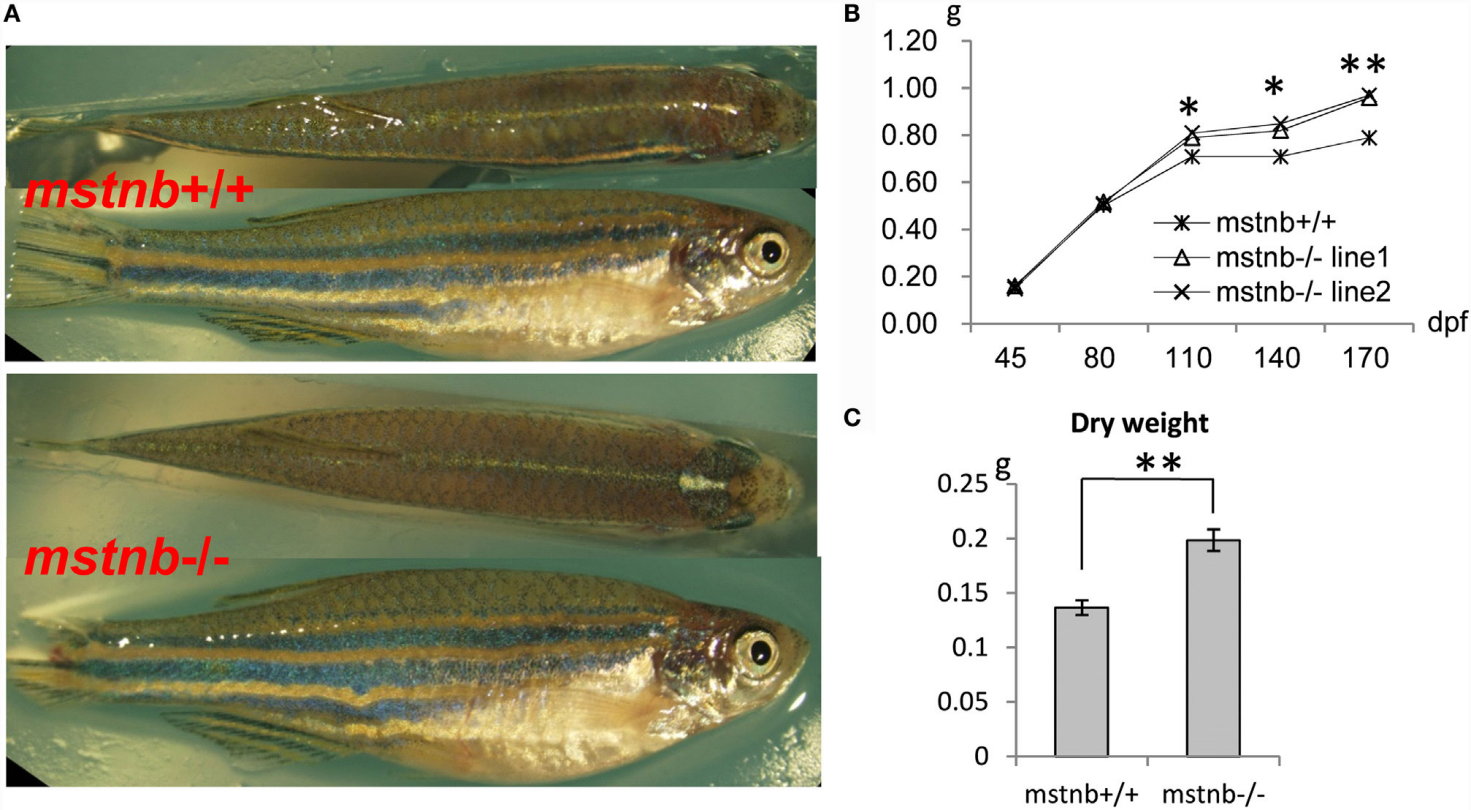
**General growth characterization of *mstnb-*deficient zebrafish**. **(A)** Wild-type zebrafish (top panel) and *mstnb-*deficient zebrafish (bottom panel) both at 100 dpf. **(B)** Curves of the somatic growth of *mstnb*-deficient zebrafish and wild-type control from the juvenile stage to the adult stage. **(C)** Dry weight of *mstnb*−/− and wild-type zebrafish at 120 dpf stage. * and ** indicate significant differences at (*P* < 0.05) and very significant differences at (*P* < 0.01), respectively. Up to 55 individuals of *mstnb*-deficient zebrafish and 50 individuals of wild-type control were checked in the experiments and the data were consistent.

**Figure 3:**
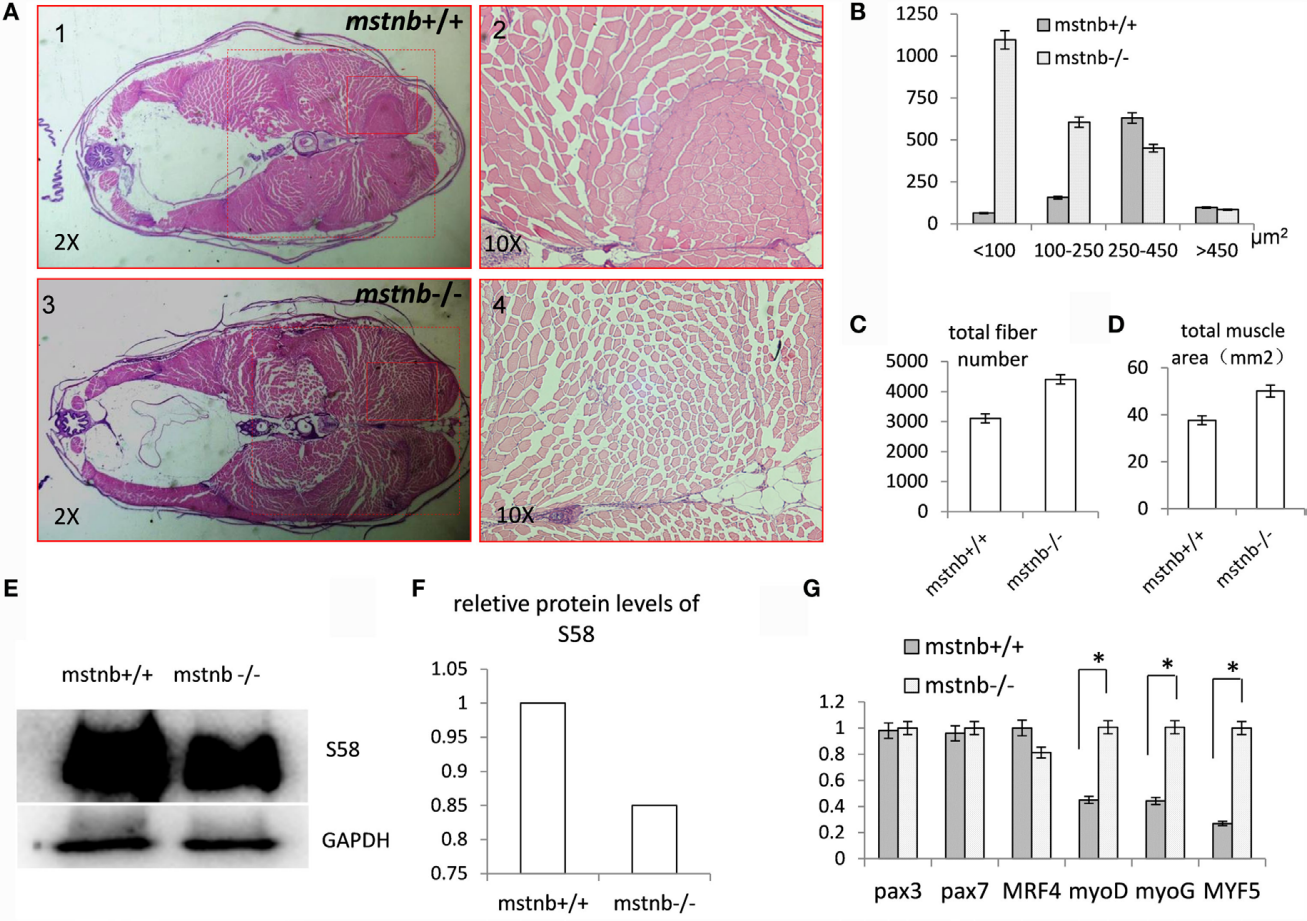
**General characterization of the muscle tissues of *mstnb-*deficient zebrafish**. **(A)** Paraffin section and H&E staining of *mstnb*-deficient zebrafish (A3, A4) and wild-type control fish (A1, A2). Lower magnification images are shown in A1 and A3; higher magnification of the indicated regions of A1 and A3 are shown in A2 and A4. **(B)** The distribution of muscle numbers are according to theirs sizes. The Y-axis indicates the numbers of muscle fibers at certain cross areas (μm^2^) of <100, 100–250, 250–450, and >450. The dotted rectangles in A1 and A3 define the areas for analysis. **(C)** The total fiber number of *mstnb*−/− and *mstnb*+/+ zebrafish sections. **(D)** The diagram shows the total area of muscle tissue in the body cross-section. **(E)** Western blot analysis of slow myofiber-specific protein S58 from muscle tissue of *mstnb*+/+ (left panel) and *mstnb*−/− (right panel). GAPDH protein was used as an internal control. **(F)** The western blot analysis was quantified by gray value analysis using Image J software. **(G)** Transcriptional expression levels of myogenesis genes. * indicate significant differences at (*P* < 0.05).

Homozygous *mstnb*-deficient zebrafish were raised with wild-type siblings derived from the same F1 heterozygous mating in the same tank. The sequence of the *mstnb* locus of tail genomic DNA served as a distinguishing genotyping marker. The body weight and body length of the two *mstnb* mutant line were examined periodically. Homozygous *mstnb*-deficient zebrafish began to exhibit 21% of weight increase compared with their control siblings after 80 dpf (Figures 2A–C). However, no obvious difference in body length between *mstnb*-deficient fish and wild-type controls was observed (data not shown). Therefore, the phenotype was specifically contributed by *mstnb* depletion. The histological examinations through sectioning and H&E staining indicated that the numbers of the muscle fibers in the *mstnb*-deficient zebrafish increased dramatically with smaller areas compared with the wild-type control fish (Figures 3A–D), suggesting that the excessive muscle growth was due to myocyte hyperplasia and not due to hypertrophy that had been shown in mammalian models (5, 6). Because significant muscle growth was promoted, we wondered whether the muscle fiber type composition had been affected. The slow MYHC-specific Marker S58 was studied using western blot analysis. The protein extracted from major muscle tissue from an *mstnb*-deficient and wild-type control fish were separated by SDS-PAGE gel and subjected to western blot assay with s58 antibody and GAPDH antibody and load same amount in the gel. The S58 protein level was slightly decreased in the *mstnb*-deficient zebrafish compared with the wild-type controls (Figures 3E,F). To determine if *mstnb* depletion enhanced the expression of myogenesis genes, the transcriptional levels of *myoD, myoG*, and *Myf5* were analyzed through quantitative PCR. The expression levels of *myoD, myoG*, and *Myf5* in *mstnb*-deficient fish muscle were significantly upregulated by 2.27-, 2.15-, and 3.71-fold compared with the wild-type controls at the adult stage, respectively (Figure 3G). However, the expression levels of *pax3* and *pax7*, two of the key regulators for muscle satellite cell proliferation (25, 26), remained at similar levels for both *mstnb*-deficient fish and wild-type zebrafish at the adult stage. Furthermore, the expression levels of *Mrf4*, which regulates myogenesis and muscle regeneration, were determined, and no significant difference in transcriptional expression were found between *mstnb*-deficient and wild-type controls at the adult stage between. Therefore, the increased muscle growth was not caused by adult satellite cell activation.

### Depletion of *mstnb* Caused Increased Serum Glucose Levels and Ectopic Accumulation of Adipose Tissue in Zebrafish Muscle

**Figure 4:**
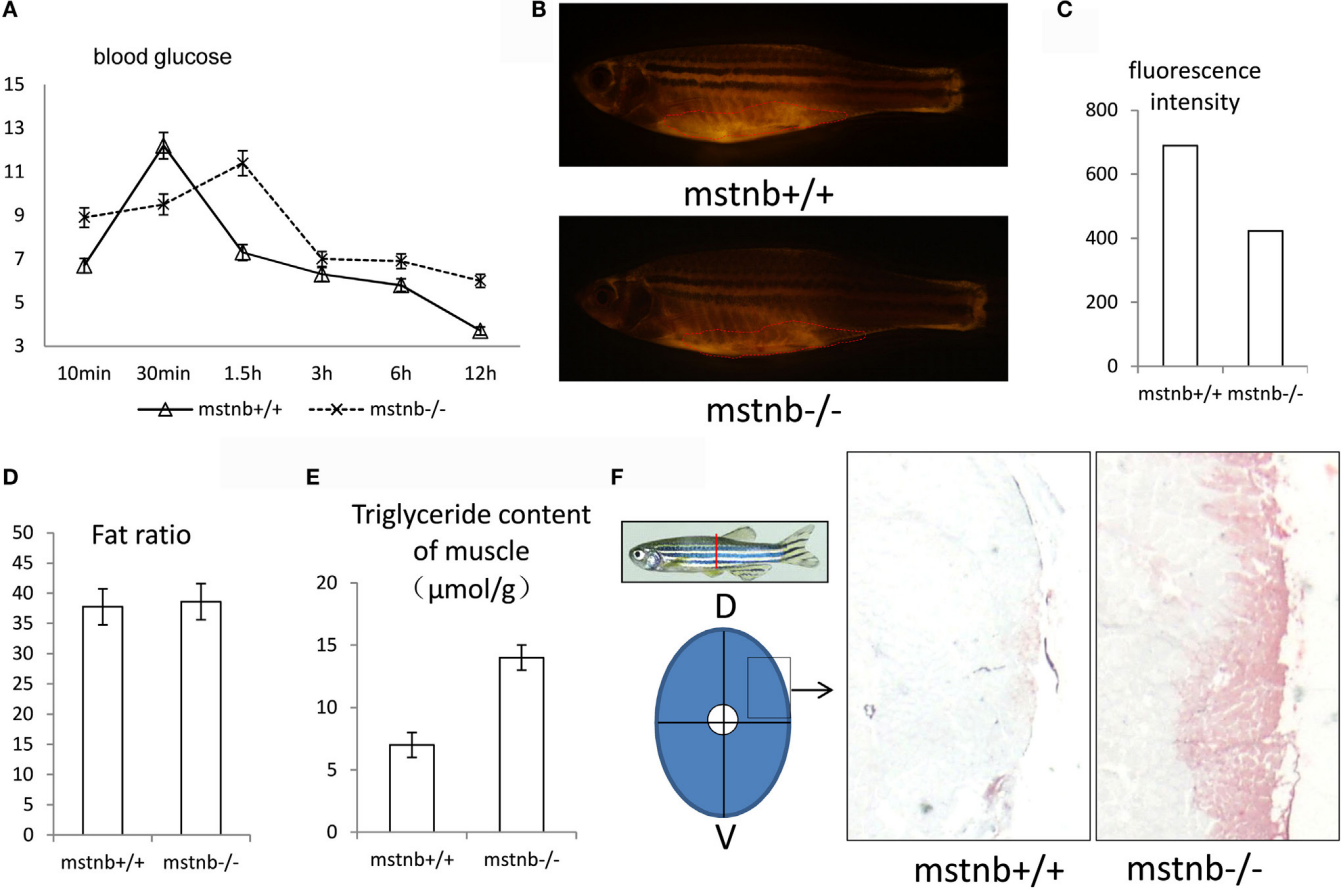
**General features of glucose utilization and fat distribution in *mstnb*-deficient zebrafish**. **(A)** The dynamic serum glucose levels of zebrafish after a chow diet. The solid line represents wild-type control zebrafish, and the dotted line represents *mstnb*-deficient zebrafish. **(B)** Representative fluorescent image of adipose tissue of a wild-type control zebrafish (upper panel) and an *mstnb*-deficient zebrafish (lower panel) at the adult stage (90 dpf) stained with Nile red under an excitation wave length of 470 nm. A marked decrease in the intensity of the subcutaneous adipose tissue staining (indicated by dotted curve) was observed in the *mstnb-*deficient adults (bottom panel) compared with the wild-type control fish (upper panel). **(C)** The quantification analysis of fluorescence intensity of Nile Red staining using Image J software. **(D)** No significant difference in body fat ratio (total fat/body weight) was observed between *mstnb-*deficient and wild-type control zebrafish. **(E)** TG content analysis of skeletal muscle indicated elevated accumulation of TG. Five pairs of zebrafish were used for analysis. The skeletal muscle tissue was dissected in the same position, and the muscle tissue was weighed. **(F)** Increased fat accumulation in the muscle tissue of *mstnb-*deficient zebrafish compared with wild-type control fish. Diagram on the left panel shows the location of muscle tissue for cross-cryosectioning and Oil red O staining of muscle tissue from adult zebrafish in the right panel. D, dorsal side; V, ventral side.

In view of the physiological phenotypes regarding carbohydrate and lipid homeostasis in mstn-null mice reported previously (8), the blood glucose dynamics in the *mstnb*-deficient zebrafish were evaluated. As the results showed in Figure 4A, the fasting glucose levels of the *mstnb*-deficient zebrafish were generally elevated with a postponed peak time compared with the control fish. However, from the peak point, the blood glucose levels recovered to normal in a shortened time frame compared with the wild-type control fish. This finding suggested that glucose utilization and anabolic metabolism were elevated. However, in the fasting state, the blood glucose concentration increased in the *mstnb*-deficient zebrafish compared with the wild-type control fish. This result may be caused by gluconeogenesis, which will be demonstrated in the next section. Meanwhile, the overall adipose tissues in zebrafish were examined through whole mount Nile red staining and diethyl ether extraction. It has been shown that the sizes of the subcutaneous and VAT in *mstnb*-deficient zebrafish decreased significantly compared with wild-type zebrafish (Figures 4B,C). However, no significant difference in the total fat/total body weight ratios between *mstnb*-deficient fish and control fish was observed (Figure 4C). To clarify this contradiction regarding the fat content, fat in muscle tissue was further tested (Figure 4D). Assays of skeletal muscle triglycerides and Oil Red O staining of skeletal muscle sections indicated a high accumulation of lipids in the muscle tissue of *mstnb*-deficient zebrafish compared with those of control fish (Figures 4E,F). This observation indicated that extra lipids tend to be transferred into skeletal muscle tissue from conventional fat deposit sites due to the *mstnb* depletion.

### Enhanced Lipid Metabolism in Muscle and the Transition of Energy Metabolism to a Lipid-Dependent Manner in *mstnb*-Deficient Zebrafish

**Figure 5:**
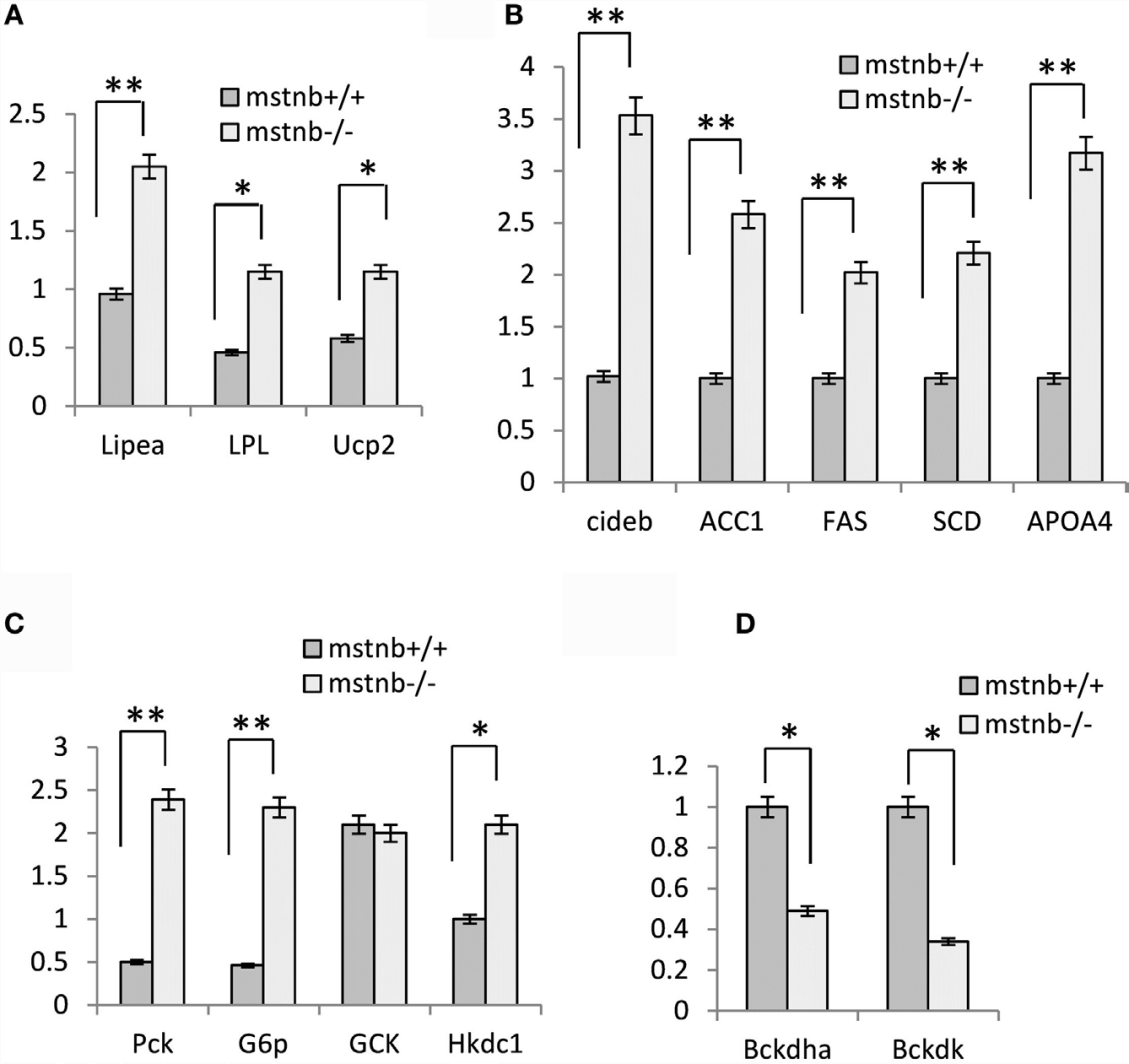
**The transcriptional levels of key molecules in the metabolic process assayed with real-time PCR**. RNA samples were extracted from muscle of *mstnb*-deficient fish and wild-type control fish at 90 dpf. The real-time RT-PCR was performed with specific primers listed in Table 1, and the relative transcript levels were determined by real-time RT-PCR using *EF-1*α as the internal standard. The expression levels of the genes in the muscle were assayed with samples from both the M1 and M2 mutant lines. **(A)** Genes involved in lipolysis; **(B)** genes involved in lipogenesis; **(C)** genes involved in gluconeogenesis and glycolysis genes; and **(D)** genes involved in amino acid degradation. * and ** indicate significant differences at (*P* < 0.05) and very significant differences at (*P* < 0.01) with at least an onefold difference, respectively. The experiments have four repeats, and the results were consistent with each other.

To further characterize the metabolic features in the muscle tissue of the *mstnb*-deficient zebrafish, the expression profiles of several key enzymes and molecules involved in lipolysis, lipogenesis, gluconeogenesis, glycolysis, and protein catabolism processes were analyzed. *Lipase-hormone sensitive a* (*Lipea*) expressed in adipose tissue and skeletal muscle hydrolyzes stored triglycerides to free lipid acid for catabolism (27, 28). Lipoprotein lipase (LPL) has the dual functions of triglyceride hydrolase and ligand/bridging factor for receptor-mediated lipoprotein uptake (29). Mitochondria uncoupling protein 2 (Ucp2), which burns lipids as heat, is commonly expressed in mammalian BAT and in multiple tissues of zebrafish (Figure 5A) (30–32). The *Lipea, LPL*, and *Ucp2* mRNAs were upregulated significantly in muscle, indicating elevated activity of lipolysis in muscle tissue. *Acetyl-CoA carboxylase* (*ACC1*) and *fatty acid synthase coding gene* (*FAS*), which are involved in lipogenesis, were transcriptionally elevated (Figure 5B) (33, 34). Cell death-inducing DFFA-like effector b (*Cideb*) promotes lipogenesis and adipocyte development (35–37). In the muscle tissue of *mstnb*-deficient zebrafish *cideb* and *stearoyl-CoA desaturase* (*SCD*) expression showed upregulated (Figure 5B). This finding indicated improved activity of lipogenesis in the muscle of *mstnb*-deficient zebrafish. ApoA4 (apolipoprotein A4) has been ascribed a wide variety of functions in lipid metabolism and metabolic regulation (29, 38, 39). The most notable characteristic of *ApoA4* is the close association with intestinal lipid absorption and bulk lipid transport. In cultured pig intestinal epithelial cells, transfection of *Apoa4* strongly enhanced transcellular TG transport. In the muscle tissue of *mstnb*-deficient zebrafish, the expression of *ApoA4* was upregulated up to threefold. This finding suggested that increased transcellular TG transport occurred (Figure 5B). The expression of *Phosphoenolpyruvate carboxykinase 1* (*PCK1*) and *glucose-6-phosphatase c family a1* (*G6pca1*), rate-limiting enzymes of gluconeogenesis (4), was significantly upregulated in the muscle tissue of *mstnb*-deficient zebrafish (Figure 5C), suggesting that gluconeogenesis processes were activated in *mstnb*-deficient zebrafish. This finding supports the increase in fasting serum glucose levels in *mstnb*-deficient zebrafish compared with the wild-type control zebrafish. However, moderate upregulation of glycolysis involved genes, such as *glucose kinase* (*GCK*) and *hexokinase domain containing 1* (*Hkdc1*), was detected in the muscle of *mstnb*-deficient zebrafish compared with the wild-type control fish (Figure 5C). *Branched-chain ketoacid dehydrogenase kinase* (*Bckdk*), *branched-chain keto acid dehydrogenase E1* and *alpha polypeptide* (*Bckdha*) are members of the protein kinase family and serve as activators of valine, leucine, and isoleucine catabolic pathways (40). *Bckdk* and *Bckdha* were transcriptionally activated in the muscle of *mstnb*-deficient zebrafish compared with the wild-type control fish (Figure 5D), suggesting a decrease in amino acid degradation. In summary, a transition of energy supply was observed from an amino acid-dependent source to a lipid-dependent source.

## Discussion

Myostatin is one member of the TGFβ superfamily that plays a principle role as a negative regulator of myogenesis and muscle growth (5, 6). Previous studies showed that a mutation of MSTN caused a double-muscled phenotype in cattle and mice. The muscle cells are characterized by both hyperplasia and hypertrophy morphology (5, 6). In this study, *mstnb*-depleted zebrafish lines were generated using TALENs techniques. We checked the *mstna* and *mstnb* expression in zebrafish muscle. The results indicated that little compensation effects of *mstna* were observed. No difference in body weight between the *mstnb*-deficient zebrafish and control zebrafish was observed until 80 dpf. However, after 80 dpf, the body weight of *mstnb*-deficient zebrafish exceeded the wild-type control zebrafish (Figure 2). *Mstnb*-deficient zebrafish showed increased muscle fiber hyperplasia at the adult stage. The sizes of the muscle fibers in the *mstnb*-deficient zebrafish decreased significantly compared with the wild-type control fish. Unlike the hypertrophied myocytes observed in the rodent models, the growth of the muscle mass in zebrafish seemed to be more dependent on myoblast proliferation due to the loss of the MSTNb functions. Our histological examinations through sectioning and H&E staining (Figure 3) indicated that excessive muscle growth was due to myocyte hyperplasia, but not due to hypertrophy as observed in mammalian models (5, 6). Previously, overexpressing the MSTN prodomain and MSTN-interacting protein follistatin in zebrafish muscle tissue resulted in myocyte hyperplasia, but not hypertrophy (9, 38). Overexpression of the dominant-negative form of *myostatin* resulted in doubling of muscle fiber number in transgenic medaka (13). In this laboratory, a growth-enhanced SOCS1a-deficient zebrafish experienced myocyte hyperplasia and hypertrophy as well (4). The enhanced somatic growth of fish, especially in the small fish model such as zebrafish, mainly resulted from myocyte hyperplasia; however, hypertrophy also has been generally recognized (4, 13, 38, 41). The observed myocyte hypertrophy in the double-muscle phenotype of the *mstn* antisense RNAi transgenic zebrafish reported by Lee et al. (12) was an exception. The antisense overexpressing model can only cause the partial depletion of the MSTN function in its model. As in all of the transgenic models, the site of the insertion of the overexpressing vector in the genome always should be a concern for the effects on its phenotypes. The complete and specific depletion of MSTN based on the TALENs approaches in our current *mstnb*-deficient model can effectively surmount such potential faults. However, the transcriptional expression levels of *pax3* and *pax7* remained similar between *mstnb*-deficient zebrafish and wild-type control zebrafish. Whether the functions of the MSTNb in zebrafish are involved in the inhibition of the renewal of satellite cells or involved in the promoting of myocyte apoptosis requires further clarification.

Myostatin depletion leads to the suppression of body fat accumulation resulting in lean mice (7). Loss of MSTN could drive the burning of WAT in mice (8). Thus, the body fat mass was suppressed and decreased significantly (7). In zebrafish, as a poikilothermic animal, BAT was not present. Lipolysis activity could not take place to induce brown adipogenesis as it does in mammals. Mitochondria are rich in muscle tissue in order to get enough energy for muscle contraction (42). Therefore, fish muscle tissue is an important site for lipolysis (43) and gluconeogenesis (44, 45). Muscle tissue may contribute to lipolysis and thermogenesis similar to the brown or beige adipose tissue in mammals. Interestingly, less subcutaneous and VAT were observed in the *mstnb*-deficient zebrafish compared with wild-type control fish, while evident lipid accumulation in the muscle tissue was observed in the *mstnb*-deficient fish (Figure 4).

Muscle mass accounts for a larger proportion of the body mass in fish compared with mammals. In our present studies, the enhanced muscle growth resulted in an increase in body weight, which would be an integrated consequence of transformed somatic metabolism due to *mstnb* depletion. To elucidate the mechanism of the energy metabolism features in the *mstnb*-deficient fish, we measured the expression of key genes in the muscle tissue using quantitative PCR. According to our results (Figure 5), the expression levels of the genes involved in lipogenesis, lipolysis, and gluconeogenesis were elevated in the *mstnb*-deficient zebrafish, while the expression levels of the genes involved in branched amino acid degradation decreased in the mutant fish. This finding suggested that lipid metabolism became highly activated, indicating that more energy was derived from lipids in the muscle tissue of *mstnb*-deficient zebrafish. In the muscle tissue of *mstnb*-deficient zebrafish, the expression of *ApoA4* was upregulated up to threefold. This finding suggested that increased transcellular TG transport occurred (Figure 5B). More lipids were transported from body fat tissue to the muscle in *mstnb*-deficient zebrafish compared with wild-type control. Therefore, the lipolysis process was enhanced and the excess fatty acid that lacks oxidative capacity would be synthesized to fat. It was consistent with the increased lipid content in muscle tissue and the increased expression of *Cideb*. Therefore, *mstnb*-deficient zebrafish adapt the preference to lipids as an energy source for enhanced somatic growth and anabolic metabolism. To sum up, the *mstnb*-deficient lines were of great value in muscle growth regulation study and mechanisms of metabolic regulation.

## Author Contributions

ZD performed the zebrafish targeting and YG and QL performed most other works. CS, GZ and XJ helped in maintaining the fish and also helped in some sampling. JH supervised the training and modified the draft. QL wrote and modified the draft. ZY initiated and supervised the research team and wrote the paper.

## Conflict of Interest Statement

The authors declare that the research was conducted in the absence of any commercial or financial relationships that could be construed as a potential conflict of interest.
