# Corrigendum: Depletion of Myostatin b Promotes Somatic Growth and Lipid Metabolism in Zebrafish

**DOI:** 10.3389/fendo.2019.00332

**Published:** 2019-05-22

**Authors:** Yanping Gao, Ziru Dai, Chuang Shi, Gang Zhai, Xia Jin, Jiangyan He, Qiyong Lou, Zhan Yin

**Affiliations:** ^1^Key Laboratory of Aquatic Biodiversity and Conservation, Institute of Hydrobiology, Chinese Academy of Sciences, Wuhan, China; ^2^University of Chinese Academy of Sciences, Beijing, China; ^3^Key Laboratory of Development and High-Value Utilization of Beibu Gulf Seafood Resource, Guangxi Key Laboratory of Beibu Gulf Marine Biodiversity Conservation, College of Food Engineering, Qinzhou University, Qinzhou, China

**Keywords:** zebrafish, myostatin, myogenesis, lipogenesis, energy metabolism

In the published article, there was an error in affiliation 2. Instead of “University of Chinese Academy of Science, Beijing, China,” it should be “University of Chinese Academy of Sciences, Beijing, China.”

The authors apologize for this error and state that this does not change the scientific conclusions of the article in any way. The original article has been updated.

